# *Listeria innocua* infection in an old case of total knee replacement – an unusual case report

**DOI:** 10.1099/acmi.0.000524.v3

**Published:** 2024-01-17

**Authors:** Minakshi Gupta, Uma Shankar Saha, Ritesh Kumar, Jayanta Laik, Minakshi Mishra

**Affiliations:** ^1^​ Department of Pathology, Tata Main Hospital, Jamshedpur, Jharkhand, India; ^2^​ Department of Joint Replacement and Orthopaedics, Tata Main Hospital, Jamshedpur, Jharkhand, India

**Keywords:** *Listeria innocua*, prosthetic implant infection, total knee replacement

## Abstract

Prosthetic implant-associated arthritis due to *Listeria* is mostly reported for *Listeria monocytogenes*. Here, we describe a patient who underwent total knee replacement 12 years ago and presented with pain, tenderness, redness and local rise in temperature in the right knee. Purulent fluid was aspirated. Upon microbiological analysis, culture yielded *Listeria innocua*. *L. innocua* is rare. *Listeria* is not reported as a contaminant and routine cultures may be negative. Because of the long interval between surgery and the onset of symptoms, clinical suspicion, radiological investigations and analysing multiple samples are of immense help.

## Data Summary

No new data have been generated.

## Introduction

*Listeria*, a Gram-positive bacillus, is believed to be pathogenic and is associated with invasive diseases such as foodborne listeriosis, meningitis and bacteraemia, usually in immunocompromised individuals [1]. *Listeria* is ubiquitously present in moist environments, soil, water and decaying vegetation. Bone and joint infection due to *Listeria* are quite rare and are mostly reported for *Listeria monocytogenes* [2, 3]. Another species, *Listeria innocua*, has even more rarely been associated with human disease [4]. We report a case of joint infection due *to L. innocua* in an old case of total knee replacement.

## Case presentation

An older female patient with a 3 month history of right knee discomfort, redness and oedema reported to the orthopaedic outpatient department (OPD). This patient has been a known diabetic for the last 20 years with fair blood sugar management. Local examination revealed swelling of the right knee with mild erythema and local rise of temperature over the right knee, which was tender on palpation with slight restriction in movement. Twelve years previously, the patient underwent bilateral total knee replacement (TKR) surgery. Before this occurrence, there was no prior history of the same symptoms after surgery. Blood test results showed TLC 12 000 mm^−3^, ESR 82 mm first hour and CRP 68 mg dl^−1^. No abnormality was seen on the AP or lateral X-ray of either knee. Cefuroxime 500 mg twice daily was started empirically with significant response. After 15 days, blood tests showed a >6× drop in ESR (12 mm first hour) and an 18× drop in CRP (3.74 mg dl^−1^). With this, the patient again presented to OPD after 3 weeks with mild pain and swelling. On examination there was mild tenderness over the right knee joint line with mild effusion but no erythema. Thick straw-coloured turbid fluid was aspirated and subjected to cytological, biochemical and microbiological analysis. ESR and CRP were also raised again to 101 mm first hour and 12.18 mg dl^−1^, respectively.

**Fig. 1.**
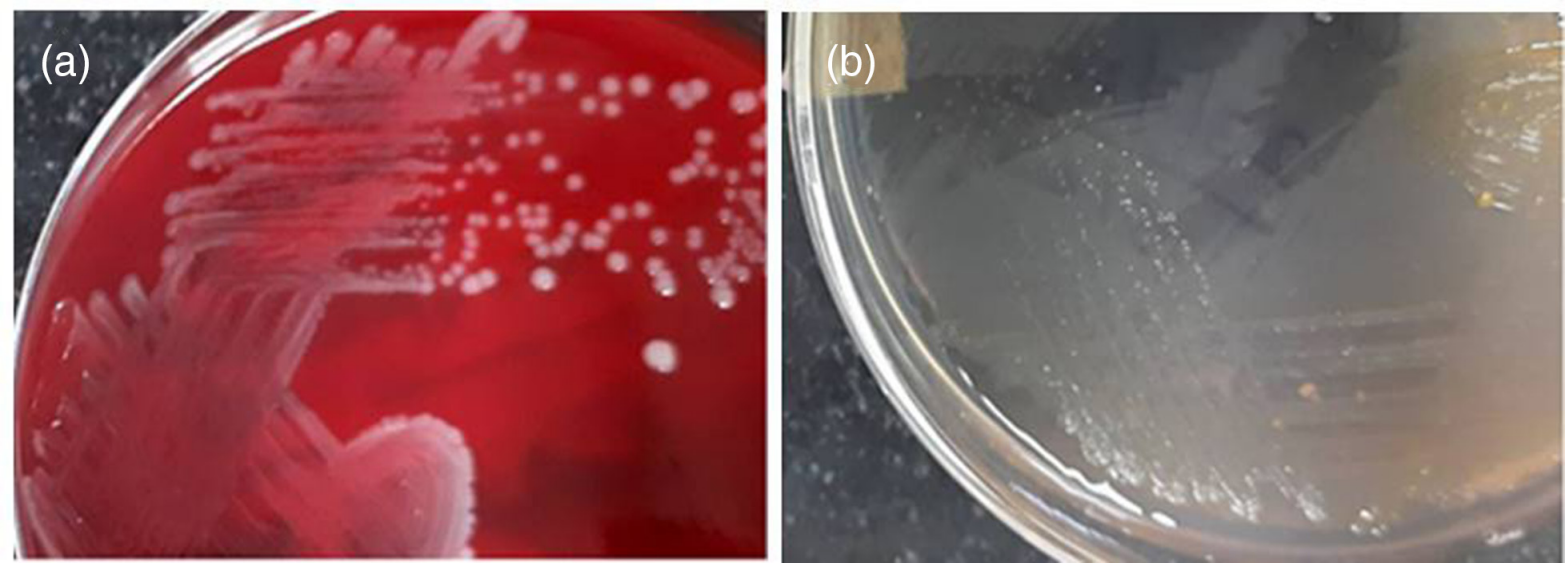
Colonies of *Listeria innocua* on (**a**) blood agar and (**b**) cystine lactose electrolyte-deficient (CLED) agar.

On fluid analysis, the total cell count was 26000 mm^−3^ with predominant polymorphonuclear cells, high LDH 1451.7 U l^−1^ with protein 6.3 gm dl^−1^ and no micro-organism on Gram stain. The fluid was inoculated in a Bact Alert FA bottle (bioMérieux, Inc., USA) and incubated in Bact Alert, which is an automated blood culture incubation instrument, at 37 °C and showed positive growth after 2 days of aerobic incubation. Upon subculture, growth revealed non-haemolytic, non-lactose-fermenting fine colonies of Gram-positive rods (Fig. 1). This was identified as *L. innocua* by VITEK 2 (bioMérieux, Inc., USA) and found to be susceptible toward extended-spectrum penicillin, aminoglycosides, macrolides and fluoroquinolones.

The patient was treated conservatively with a combination of ampicillin, 500 mg, 6 hourly, and gentamicin, 1 mg kg^−1^, IV for 2 weeks. On the first follow-up, which was 13 days after the initiation of antibiotics, ESR and CRP had fallen to 71 mm first hour and 4.26 mg dl^−1^, respectively, which further decreased to 58 mm first hour and 1.19 mg dl^−1^, respectively, at the second follow-up after 35 days (Table 1). The patient also improved symptomatically and on follow-up there was no recurrence.

**Table 1.** Timeline of patient follow-up and blood test results

2009	12 December 2021	30 December 2021	23 January 2022	30 January 2022	12 February 2022	17 March 2022
TKR	ESR, 82 mm first hour; CRP, 68 mg dl^−1^; Cap cefuroxime stat	ESR, 12 mm first hour; CRP, 3.74 mg dl^−1^	ESR, 101 mm first hour; CRP, 12.18 mg dl^−1^; symptoms reappeared	Culture positive for *Listeria innocua*; antibiotics revised	ESR, 71 mm first hour; CRP, 4.26 mg dl^−1^	ESR, 58 mm first hour; CRP, 1.19 mg dl^−1^

## Discussion

Gram-positive cocci such as *Staphylococcus* and coagulase-negative *Staphylococcus* are the most important aetiological agents for hip and knee prosthetic and periprosthetic joint infections, accounting for 50–60 % of cases. Gram-negative bacteria and other atypical organisms are now recognized as causative agents in immunocompromised individuals, with an upsurge in joint replacements [5, 6]. One such unusual agent is the genus *Listeria* in which *Listeria monocytogenes* is usually associated with rare bacterial diseases such as listeriosis, caused by contaminated food stored for long periods at a low temperature, sepsis and meningoencephalitis. This can cause serious illness in pregnant women, neonates, the elderly and immunocompromised individuals. The incubation period varies from a few days to 3 months.

Listeriosis on rare occasions may manifest as local infections in different organs such as bones and joints [7]. It may lead to septic arthritis involving prosthetic devices. The first case was reported way back in 1980 by Neiman and Lorber [8]. Endocarditis associated with prosthetic valves, endophthalmitis and lymphadenitis were also reported [9–14]. Due to the unusual nature of the bacterium, its rarity of isolation and identification and the relatively lengthy time between surgery and the onset of symptoms, the association between *Listeria* and arthritis is less well known. Sometimes, symptoms may not be that evident or incapacitating because of latent or subclinical infection. Case series and isolated reports have been published about *L. monocytogenes* with prosthesis, but there are only two case reports (one of bacteraemia and a second for neonatal meningitis) involving *Listeria innocua*, reported in 2003 and 2014 [15, 16]. To the best of our knowledge, this is the first report for a case of prosthetic joint infection due to *L. innocua*.

This case study is unique in describing a rare aetiology for an infection in a very old case of a total knee replacement. No revision surgery or implant removal was required, since the patient was managed conservatively and responded to antibiotics. It probably presented as an indolent low-grade infection.
